# Clinical History and Burden of Gaucher Disease Type 3: A Cross‐Sectional Multinational Study

**DOI:** 10.1002/jmd2.70127

**Published:** 2026-10-30

**Authors:** Eugen Mengel, Sinziana Stanescu, Alessio Di Fonzo, Aimee Donald, Tanya Collin‐Histed, Laurence Djatche, Idaira Rodriguez Santana, Jagan Mohan Palli, Maria Gabriela Perichon, Ruth Pulikottil‐Jacob, Fatih S. Ezgu

**Affiliations:** ^1^ SphinCS Hochheim Germany; ^2^ Pediatrics, Hospital Universitario Ramon y Cajal Madrid Spain; ^3^ Fondazione IRCCS Ca' Granda Policlinico Hospital of Milan Milan Italy; ^4^ University of Manchester Manchester UK; ^5^ International Gaucher Alliance (IGA) London UK; ^6^ Sanofi Bridgewater Massachusetts USA; ^7^ Prime HCD Knutsford UK; ^8^ Sanofi Hyderabad India; ^9^ Global Medical Director Rare Hematology, Sanofi Cambridge Massachusetts USA; ^10^ Sanofi Reading UK; ^11^ Pediatric Metabolic and Genetic Disorders, Gazi University Hospital Ankara Turkey

**Keywords:** caregivers, disease burden, Gaucher disease Type 3, health resources, quality of life, rare diseases

## Abstract

Gaucher disease Type 3 (GD3) is a rare genetic neurodegenerative disorder, characterized by heterogeneity in symptom burden, disease severity, and progression. There is limited evidence on the burden of GD3. This retrospective, cross‐sectional study estimated the burden of illness associated with GD3 using descriptive analyses of clinician‐ and patient/caregiver‐reported data from Germany, Italy, Spain, Turkey, the UK, and the US. A total of 79 patients were enrolled in the study; 39 patients and 38 caregivers completed the patient/caregiver survey. The majority of patients were male (74.7%) and under 18 years old (55.7%), with an average age at index (study enrolment date) of 17.0 years (SD = 9.0). Patients received a GD3 diagnosis at a mean age of 8.2 years (SD = 6.4). The most common first non‐neurological symptoms experienced were splenomegaly (49.4%), anemia (44.3%), and enlarged abdomen (43.0%), while the first most common neurological manifestations reported included eye movement disorders (54.4%), epilepsy (35.4%), and neurodevelopmental delay (34.2%). Approximately half of patients experienced falls (55.7%) or difficulty with personal care (53.2%). Nearly half of patients (45.6%) had at least one hospitalization, with a mean (SD) of 2.1 (1.7) hospitalizations per patient, in the 2 years pre‐index. Caregivers were predominantly female (86.8%) and provided an average of 63.3 h of care per week (SD = 40.2). They reported high work impairment (70.3%, SD = 22.0; WPAI) and reduced quality of life (CarerQoL‐7D mean = 67.0, SD = 15.8). This study characterizes the real‐world burden of GD3, providing an up‐to‐date, multinational perspective on its clinical history and impact on patients, caregivers, healthcare systems, and society.

## Key Points

- This multinational study addresses an important evidence gap by quantifying the multidimensional burden of Gaucher disease Type 3 (GD3) on patients, caregivers, healthcare systems, and society.
- GD3 is characterized by substantial and persistent central nervous system (CNS) burden, alongside systemic manifestations, contributing to impaired mobility, functioning, independence, and daily activities.
- The complex neurosystemic burden of GD3 is associated with considerable healthcare resource use and supportive care needs, including hospitalizations, ongoing clinical monitoring, assistive devices, and home adaptations.
- The impact of GD3 extends beyond patients to caregivers and families, with intensive caregiving demands affecting employment, emotional well‐being, financial circumstances, and overall quality of life.
- The persistent CNS burden underscores the need for treatments that address CNS manifestations more effectively, together with coordinated multidisciplinary care and integrated psychosocial support.

**Abbreviations**
BAER: brainstem auditory evoked response
BGL: β‐glucosidase leukocyte
CarerQoL‐7D: Care‐related Quality of Life instrument—7 Dimensions
CRF: case report form
CT: computerized tomography
DMD: Duchenne muscular dystrophy
DXA: dual‐energy X‐ray absorptiometry
EQ‐5D‐5L: EuroQol—5 Dimensions—5 Levels
GD: Gaucher disease
GD1/2/3: Gaucher disease Type 1/2/3
HRQoL: health related quality of life
ICCG: International Collaborative Gaucher Group
MRI: magnetic resonance imaging
mSST: modified severity scoring tool
nGD: neuronopathic forms of Gaucher disease
PC: patient/caregiver
PRO: patient reported outcome
QoL: Quality of Life
SALT: speech and language therapy
SD: standard deviation
UK: United Kingdom
US: United States
USS: ultrasound Scan
VAS: visual analog scale
WPAI: Work Productivity and Activity Impairment

## 1 Introduction

Gaucher disease (GD) is a rare inherited autosomal recessive chronic and progressive lysosomal storage disease caused by mutation in the *GBA* gene, which consequently causes a deficiency in acid β‐glucosidase enzymatic activity and the accumulation of glycosphingolipids, glucosylceramide [1, 2, 3, 4]. Clinically, GD is classified into three major types and two forms [5]. Gaucher disease Type 1 (GD1) is characterized by the absence of primary central nervous system (CNS) involvement. Types 2 and 3 (GD2 and GD3) affect the nervous system and are known as neuronopathic forms of Gaucher disease (nGD). GD2 usually manifests in early infancy, and GD3 can manifest at any time after birth. GD2 and GD3 differ in the severity and progression of the CNS involvement. GD2 is characterized by acute CNS manifestations, while GD3 is characterized by chronic CNS manifestations [6].

Patients with GD3 show considerable heterogeneity in symptom burden, disease severity, and disease progression. They may experience many of the same visceral manifestations observed in GD1, including enlargement of the liver and spleen, anemia, and thrombocytopenia, while also exhibiting heterogeneous presentation of CNS involvement [4, 7, 8]. Importantly, beyond neurosystemic manifestations, skeletal involvement (e.g., osteonecrosis, bone crises, and bone deformities) is also common in GD3 and contributes substantially to morbidity and healthcare resource utilization [9]. Pulmonary manifestations, including interstitial lung disease, may also occur and can affect prognosis and quality of life [5, 10].

GD3's rarity and heterogeneity can cause delays between symptom onset and diagnosis [11, 12]. Recent real‐world evidence from the International Collaborative Gaucher Group (ICGG) Gaucher Registry further highlights the variability in clinical presentation among children with GD3. These data also indicate that early initiation of imiglucerase in pediatric patients with GD1 and GD3 is associated with sustained improvements across hematological, visceral, growth, and skeletal outcomes, underscoring the importance of early diagnosis and timely treatment [13]. Most patients with GD3 are diagnosed in childhood [12, 14, 15, 16, 17, 18] and, because the severity of their CNS manifestations increase over time, require the support of a caregiver [19, 20]. GD is a rare disease, with an overall prevalence (all types) of 0.9 cases per 100 000 people [21]. Data from international studies indicate that around 6%–7% of patients with GD have GD3 [22, 23]. The percentage of patients with GD who have GD3 varies across countries/regions, ranging from 4% to 11% in Latin America [24, 25] and parts of Europe [14, 15, 16, 25], to over 30% in Japan [26], Korea [27], and Turkey [28], and exceeding 40% in Sweden [29], Poland [30], Thailand [31], and Egypt [17, 32].

Given the rarity, heterogeneity, and variable prevalence of GD3 across regions, it is important to assess its multidimensional burden, encompassing the overall impact on patients, families, healthcare systems, and society.

Previous studies have assessed the overall burden of GD without distinguishing between GD types, showing that the disease can lead to large healthcare expenditure [33, 34, 35, 36, 37] and negatively affect caregivers' quality of life [34, 38]. However, these aggregate assessments do not adequately capture the distinct clinical complexity and lived experience of GD3, for which evidence remains limited, particularly on healthcare resource use, patient‐reported outcomes (PROs), and caregiver burden.

The aim of this study was to address a gap in knowledge by characterizing the neurosystemic burden of GD3 across multiple countries, highlighting its impact on patients, caregivers, healthcare systems, and society. This was achieved by documenting patients' clinical history, diagnostic journey, symptoms, impact on daily life, and healthcare resource utilization. Additionally, the study sought to quantify caregiver burden by assessing health‐related quality of life and work productivity impairment across six countries: Germany, Italy, Spain, Turkey, the UK, and the US.

## 2 Data and Methods

### 2.1 Study Design

This was an observational, descriptive, retrospective, international study with cross‐sectional data collection, designed to collect real‐world data on the burden of illness associated with GD3.

### 2.2 Data Collection

Patient‐level data were collected during 2023 to 2024 from clinicians who were recruited via physician panels in Germany, Italy, Spain, Turkey, UK, and US. Clinicians practicing the following specialties were invited to participate: neurology, ophthalmology, pulmonology, pediatrics, hematology, hemato‐oncology, inherited metabolic disease, internal medicine, hepatology, gastroenterology, genetics, physical therapy and rehabilitation, and orthopedics.

Patients diagnosed with GD3 at least 12 months prior to the index date were invited to participate in the study by clinicians currently managing their care, using a “next suitable patient seen” or prospective random selection approach. The index date was defined as the date of the routine clinical consultation during which the patient agreed to participate in the study. Clinicians were asked to complete an online case report form (CRF) for their current patients with GD3 who participated in the study, providing information on each patient's sociodemographic characteristics, diagnosis history, clinical burden (e.g., type of symptoms, impact on daily activities), and healthcare service use. Clinicians were asked to collect data regarding the CNS and systemic manifestations that their patients reported at the index date; additionally, they collected data on the first manifestations reported by the patients and the age at which these presented for the first time.

Two measures of the impact of GD3 on daily activities were included in the CRF: falls and problems with personal care.

Clinicians invited their current patients with GD3 and their caregivers to complete an online patient/caregiver survey (PC survey), which included two sections: one focused on the patient and the other on the caregiver. For pediatric patients, as well as adult patients unable to complete the survey independently, the caregiver was asked to provide consent and complete the survey as a proxy respondent on the patient's behalf. The patient section collected data on patient characteristics and medical (e.g., consultations with specialists, procedures) and nonmedical (e.g., home alterations, alternative therapies) resource use. The caregiver section collected data on caregiver characteristics, hours spent providing care, and indirect resource use (work productivity), and caregiver QoL was assessed using caregiver‐reported outcome questionnaires (see Section 2.2.1). Results include only informal caregivers, as the single contracted caregiver (from Spain) was excluded from the analysis.

Clinicians and patient/caregivers were compensated for their time spent participating in the study. The clinician remuneration system is based on country, specialty, and length of interview, following the principle of fair market value. Patients and caregivers were offered an Amazon voucher as a token to compensate for the time and effort required to participate in the study.

#### 2.2.1 Caregiver Reported Outcomes

Caregivers were asked to complete the Work Productivity and Activity Impairment (WPAI) [39], the Care‐related Quality of Life (CarerQoL) [40], and the 5‐level EuroQol‐5 Dimension version (EQ‐5D‐5L) [41] questionnaires as part of the caregiver section of the PC survey.

The WPAI questionnaire is a validated tool for assessing the impact of GD3 on caregivers' productivity and daily activities. The instrument includes six questions covering four domains: absenteeism (work time missed), presenteeism (reduced effectiveness while at work), overall work productivity loss (a combination of absenteeism and presenteeism), and activity impairment. The recall period is 7 days. Scores are reported as percentages ranging from 0 to 100, with higher scores indicating greater impairment and lower productivity [39].

The CareQoL seven‐dimension (CarerQoL‐7D) instrument measures the impact of care on informal caregivers; it has two parts: (1) CarerQoL‐7D, consisting of seven dimensions—relational problems, mental health problems, problems combining daily activities with care, financial problems, physical health problems because of providing informal care, fulfillment from caregiving, and support with lending care—each rated on three levels (no problems, some problems, and a lot of problems); and (2) a visual analog scale (VAS) measuring the caregiver's current overall happiness, ranging from 0 (completely unhappy) to 10 (completely happy) [40]. The seven dimensions can be used to calculate a total utility score (range: 0 = worst imaginable caregiving situation; 100 = best imaginable caregiving situation) applying country specific tariffs [42]; for comparability, UK tariffs were applied to all caregivers in this study.

The EQ‐5D‐5L is a generic, multi‐attribute, health‐state classification system used to assess health‐related quality of life (HRQoL) across five dimensions: mobility, self‐care, usual activities, pain/discomfort, and anxiety/depression. Respondents rate their level of impairment in each of the five dimensions using a five‐level scale: no problems, slight problems, moderate problems, severe problems, and extreme problems. The questionnaire also includes the EQ visual analog scale (EQ VAS), which records self‐rated health on a visual scale (range: 0 = the worst health you can imagine; 100 = the best health you can imagine) [41]. Health states can be transformed into a utility index (range: 0 = health state equivalent to death; 1 = perfect health) using country specific weights [43]; for comparability, this study uses a single value set for all caregivers, the English value set [44].

### 2.3 Statistical Analysis

Data were analyzed descriptively. The actual sample size was determined by the number of accessible patients with GD3 from the participating countries.

Patient‐level data was described using number of responses (*n*), mean, standard deviation (SD), median, and the 25th and 75th percentiles (*Q*1 and *Q*3) for continuous variables, and count and percentage in each category for categorical variables. No imputation of missing data was performed. All analyses were performed in Stata 18 [45].

## 3 Results

A total of 79 patients were included in the study, based on data provided by participant physicians (see Section 3.1.1 for patient characteristics). Their data was provided by 34 clinicians (see Table S1 in Supporting Information), nine (26.5%) in Turkey, seven (20.6%) each from Spain and the US, five (14.7%) in Italy, and three (8.8%) each from Germany and the UK. The most common specialties among participating clinicians were hematology (*n* = 6, 17.7%), neurology (*n* = 5, 14.7%), genetics (*n* = 5, 14.7%), hemato‐oncology (*n* = 4, 11.8%), internal medicine (*n* = 4, 11.8%), and pediatrics (*n* = 4, 11.8%).

Approximately half of the patients (39/79) and their caregivers (38/79) completed the PC survey (see Section 3.2.1 for patient characteristics and Section 3.2.3 for caregiver characteristics).

### 3.1 Clinician Reported Data

#### 3.1.1 Patients' Characteristics

Patients' characteristics collected in the CRF are reported in Table 1. Most patients were male (74.7%) and younger than 18 years (55.7%). Their mean (SD) age at index was 17.01 (9.03) years. Patients resided in Spain (*n* = 19, 24.1%), Turkey (*n* = 17, 21.5%), Italy (*n* = 13, 16.5%), the US (*n* = 12, 15.2%), Germany (*n* = 11, 13.9%), and the UK (*n* = 7, 8.9%) at the time of the study.

**TABLE 1:** Patient characteristics and diagnosis.

Total number of patients	79
Sex, *n* (%)	
Female	20 (25.3)
Male	59 (74.7)
Country, *n* (%)	
Germany	11 (13.9)
Italy	13 (16.5)
Spain	19 (24.1)
Turkey	17 (21.5)
UK	7 (8.9)
US	12 (15.2)
Specialty of the clinician^a^	
Cardiology	1 (1.3)
Genetics	10 (12.7)
Hemato‐oncology	12 (15.2)
Hematology	11 (13.9)
Hepatology	1 (1.3)
Inherited metabolic disorders	4 (5.1)
Internal medicine	11 (13.9)
Neurology	20 (25.3)
Orthopedic medicine	1 (1.3)
Pediatrics	5 (6.3)
Other (nephrology)	3 (3.8)
Current age (years)	
Mean (SD)/median (25th, 75th percentile)	17.01 (9.03) / 16 (10; 23)
Age group, *n* (%)	
Under 18 years	44 (55.7)
18 years or older	35 (44.3)
Age at GD3 diagnosis^b^ (years) (*N* = 67)	
Mean (SD)/Median (25th, 75th percentile)	8.16 (6.36)/6 (3; 13)
Test for diagnostic confirmation of GD3^c^ (*N* = 79), *n* (%)	
BGL	73 (92.4)
Molecular genetic testing	57 (72.2)
p.L483P/p.L483P^d^	26 (45.6)
p.L483P/p.R502C^d^	10 (17.5)
p.L483P/other^d^	5 (8.8)
p.R502C/other^d^	1 (1.8)
Unknown^d^	15 (26.3)
Bone marrow biopsy	24 (30.4)
Other^e^	3 (3.8)
Unknown (Exclusive answer)	1 (1.3)
Time to GD3 diagnosis (years)	
From first somatic manifestations^f^ (*N* = 61)	
Mean (SD)/Median (25th, 75th percentile)	1.41 (3.77) / 1 (0; 2)
From first CNS manifestations^f^ (*N* = 59)	
Mean (SD)/Median (25th, 75th percentile)	0.69 (3.82) / 0 (−1; 1)

Abbreviations: BGL, β‐glucosidase leukocyte; GD3, Gaucher disease Type 3; MR brain, brain MRI; MRI, magnetic resonance imaging; SD, standard deviation.

^a^ Specialty of the clinician who completed the survey. Patients might see more than one clinician, in different specialties.

^b^ Calculated as: Age at GD3 diagnosis = year in which the patient was diagnosed with GD3—year of birth.

^c^ More than one option could be selected; percentages are calculated using the total number of patients (*N* = 79) as the denominator and do not necessarily add to 100.

^d^ Percentages calculated using number of patients with molecular genetic testing (*N* = 57) as the denominator.

^e^ Free text entered after selecting “other”: MRI (*n* = 2) and MR brain (*n* = 1).

^f^ Calculated as: Time from first somatic/CNS symptoms to GD3 diagnosis = age at GD3 diagnosis—age at first somatic/CNS symptoms. Note that age at GD3 diagnosis is calculated using calendar years (see note a), while age at first somatic/CNS symptoms was reported as age. Since a person's age changes during a calendar year, when combining these two variables differences of 1 year are common.

#### 3.1.2 GD3 Diagnosis

The mean age at GD3 diagnosis (Table 1) was 8.16 (SD = 6.36) years. Most patients (*n* = 57, 72.2%) underwent genetic testing to confirm their diagnosis. Among those tested, the most frequently reported genotypes were p.L483P/p.L483P (*n* = 26, 45.6%) and p.L483P/p.R502C (*n* = 10, 17.5%). Most diagnoses were recorded within 2 years of the first somatic manifestations (mean = 1.41, SD = 3.77) and within 1 year of the first CNS manifestations (mean = 0.69, SD = 3.82).

#### 3.1.3 Clinical Manifestations

Reported CNS and somatic manifestations are shown in Figure 1, each plot shows first manifestations and manifestations at index. A detailed description of the components included within each involvement category is provided in the figure notes.

**FIGURE 1:**
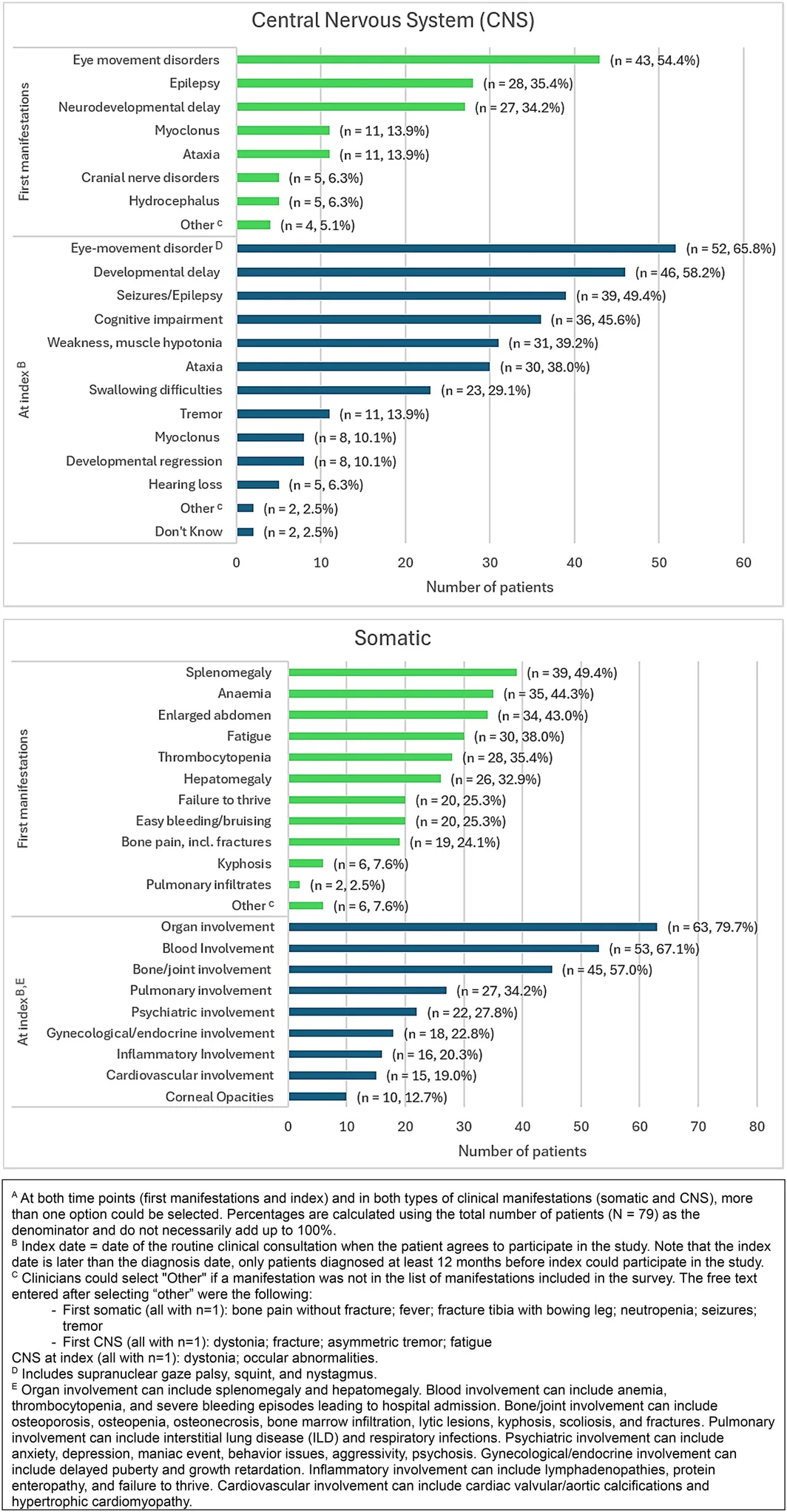
Clinical manifestations^A^.

The first CNS symptom(s)/sign(s) of GD3 manifested at a mean (SD) age of 7.3 (5.7) years (see Table S2 in the Supporting Information for age of first manifestation by symptom/sign). The most frequently reported initial CNS manifestations were eye movement disorders (which includes supranuclear gaze palsy, squint, and nystagmus; *n* = 43, 54.4%), epilepsy (*n* = 28, 35.4%), neurodevelopmental delay (*n* = 27, 34.2%), ataxia (*n* = 11, 13.9%), and myoclonus (*n* = 11, 13.9%). At index, the most frequently reported CNS manifestations were eye movement disorders (*n* = 52, 65.8%), developmental delay (*n* = 46, 58.2%), seizures/epilepsy (*n* = 39, 49.4%; *n* = 35 controlled with treatment), cognitive impairment (*n* = 36, 45.6%), and weakness, muscle hypotonia (*n* = 31, 39.2%). On average, patients with GD3 presented with three to four CNS manifestations at index (mean = 3.68, SD = 2.04).

The first somatic symptom(s)/sign(s) manifested at a mean (SD) age of 7.6 (6.8) years. The most common first somatic manifestations were splenomegaly (*n* = 39, 49.4%), anemia (*n* = 35, 44.3%), enlarged abdomen (*n* = 34, 43.0%), fatigue (*n* = 30, 38.0%), and thrombocytopenia (*n* = 28, 35.4%). At the index date, the most frequently reported somatic manifestations were organ involvement (which includes splenomegaly and hepatomegaly; *n* = 63, 79.7%), blood involvement (*n* = 53, 67.1%), bone/joint involvement (*n* = 45, 57.0%), including kyphosis (*n* = 11, 13.9%), scoliosis (*n* = 6, 7.6%), and other conditions (full list in Figure 1 footnote), pulmonary involvement (*n* = 27, 34.2%), and psychiatric involvement (*n* = 22, 27.8%).

#### 3.1.4 Impact of GD3 on Activities of Daily Living

The impact of GD3 on daily activities is shown in Table 2. Most patients have experienced falls (*n* = 44, 55.7%); around half of them (*n* = 23, 52.3%) experienced falls once or twice per month. Over half of the patients have experienced problems with their personal care (*n* = 42, 53.2%), the majority of whom experienced more than one type of problem: 97.6% of them had problems getting dressed or washed, 71.4% required feeding assistance, and 64.3% had problems going to the toilet or getting out of bed.

**TABLE 2:** Impact on daily activities.

Total number of patients	*n* (%)
	79
Number of patients who experienced falls^a^	44 (55.7)
Frequency of falls^b^	
Daily	1 (2.3)
Often (every week)	7 (15.9)
Sometimes (once or twice per month)	23 (52.3)
Hardly ever	11 (25)
Don't Know	2 (4.5)
Number of patients who experienced problems with personal care^c^	42 (53.2)
Type of difficulty^d^	
Getting dressed or washed	41 (97.6)
Feeding assistance	30 (71.4)
Going to the toilet, getting out of bed, and so on.	27 (64.3)

^a^ For nine (11.4%) patients, the clinician did not know whether the patient had experienced falls.

^b^ Percentages calculated using number of patients who experienced falls (*n* = 44) as denominator.

^c^ For four (5.1%) patients, the clinician did not know whether the patient had experienced problems with personal care.

^d^ More than one option could be selected; percentages are calculated using the total number of patients who had problems with personal care (*n* = 42) as the denominator and do not necessarily add up to 100%.

#### 3.1.5 GD3‐Related Healthcare Resource Use

Resource use associated with GD3 is reported in Table 3. Thirty‐six (45.6%) patients had at least one inpatient hospitalization in the past 2 years, accounting for a total of 75 admissions. On average, each patient had 2.1 (SD = 1.7) inpatient hospitalizations in the 2 years preceding the index date, 1.2 (SD = 1.6) in the year immediately before the index date, and 0.9 (SD = 0.8) in the year prior to that. The most reported reasons for admission were CNS (*n* = 24, 32.0%) and bone/joint (*n* = 22, 29.3%). Admissions to a general ward were the most common (*n* = 35, 46.7%), and admission to an emergency ward was the second most common (*n* = 27, 36.0%).

**TABLE 3:** Resource use.

Inpatient hospitalizations in the past 2 years^a^	
Total number of inpatient admissions	75
Type of admission, *n* (%)	
General ward	35 (46.7)
Emergency ward	27 (36.0)
Supportive care	9 (12.0)
Intensive Care Unit	4 (5.3)
Reason for admission^b^, *n* (%)	
CNS	24 (32.0)
Bone/joint	22 (29.3)
Pulmonary	15 (20.0)
Blood	10 (13.3)
Organ	9 (12.0)
Inflammatory	8 (10.7)
Ophthalmologic	3 (4.0)
Cardiovascular	2 (2.7)
Other^c^	1 (1.3)
Number of patients who had at least one inpatient admission, *n* (%)	36 (45.6)
Number of inpatient hospitalizations (*N* = 36)	
Over the 2‐year observation period; Mean (SD)/Median (25th, 75th percentile)	2.1 (1.7)/1.5 (1; 2.5)
In last year (0–12 months before index); Mean (SD)/Median (25th, 75th percentile)	1.2 (1.6)/1 (0; 1)
In second to last year (13–24 months before index); Mean (SD)/Median (25th, 75th percentile)	0.9 (0.8)/1 (0; 1)

Tests in the past 2 years^a^, ^d^, *n* (%)	
Total number of patients	79
Blood test	73 (92.4)
Neurological examination	62 (78.5)
Echocardiogram	48 (60.8)
Ocular examination	43 (54.4)
USS	39 (49.4)
Electroencephalogram	32 (40.5)
X‐ray	27 (34.2)
MRI	25 (31.6)
DXA	24 (30.4)
CT scan	16 (20.3)
Intraocular pressure test	12 (15.2)
SALT	12 (15.2)
mSST	5 (6.3)
BAER test	4 (5.1)

Health devices and home alterations^e^, ^f^, *n* (%)	
Total number of patients	39
Bathroom adaptations	25 (64.1)
Vehicle adaptations	21 (53.8)
Bed rail	21 (53.8)
Hand/foot Splints	15 (38.5)
Walker	15 (38.5)
Wheelchair manual	14 (35.9)
Walk in shower	10 (25.6)
Transfer aids	9 (23.1)
Stair lift	6 (15.4)
Cane/walking stick	5 (12.8)
Crutches	3 (7.7)
Wheelchair powered	2 (5.1)
Ramp	1 (2.6)
None (Exclusive answer)	5 (12.8)

Alternative/complementary therapies in the last 12 months^e^, ^f^, *n* (%)	
Total number of patients	39
Nutrition/diet	25 (64.1)
Physiotherapy^g^	17 (43.6)
Massage/acupuncture	11 (28.2)
Occupational therapy^g^	8 (20.5)
Swimming/aqua‐aerobics	8 (20.5)
Chiropractic	6 (15.4)
Psychology/counseling^g^	2 (5.1)
Yoga/Pilates/Tai Chi	1 (2.6)
Non‐applicable (exclusive answer)	3 (7.7)

Abbreviations: BAER, brainstem auditory evoked response; CT, computerized tomography; DEXA, dual‐energy X‐ray absorptiometry; MRI, magnetic resonance imaging; mSST, modified severity scoring tool; SALT, speech and language therapy; SD, standard deviation; USS, ultrasound scan.

^a^ Clinician‐reported data.

^b^ More than one option could be selected; percentages use the number of inpatient admissions (*N* = 75) as the denominator and do not necessarily add up to 100%.

^c^ No free text follow‐up question after selecting “other.”

^d^ More than one option could be selected; percentages are calculated using the total number of patients (*N* = 79, clinician‐reported data) as the denominator and do not necessarily add up to 100%.

^e^ More than one option could be selected; percentages are calculated using the total number of patients (*N* = 39, patient‐reported data) as the denominator and do not necessarily add up to 100%.

^f^ Patient‐reported data.

^g^ Private/non‐covered by insurance.

Patients had multiple tests in the 2 years preceding the index date; the most common were blood test (*n* = 73, 92.4%), neurological examination (*n* = 62, 78.5%), and echocardiogram (*n* = 48, 60.8%).

### 3.2 Patient/Caregiver Reported Data

#### 3.2.1 Patient Characteristics

Patients' characteristics collected in the PC survey are presented in Table 4. The PC survey was completed for around half of the patients in the study, *n* = 39/79, 49.4%. The majority of patients were male (*n* = 30, 76.9%) and younger than 18 years (*n* = 25, 64.1%). Most had either no formal education or had completed only primary education (*n* = 29, 74.4%), consistent with the predominantly pediatric profile of the study population. Their mean (SD) age at the time of the study was 15.18 (7.26) years. Patients resided in Turkey (*n* = 17, 43.6%), Spain (*n* = 11, 28.2%), Italy (*n* = 8, 20.5%), the US (*n* = 2, 5.1%), and Germany (*n* = 1, 2.6%) at the time of the study. No patients in the UK completed the PC survey.

**TABLE 4:** Patient and caregivers' characteristics^a^.

	Patients	Informal caregivers^b^
Total number of patients/caregivers	39	38
Age (years); mean (SD)	15.18 (7.26)	44.16 (9.23)
Age group, *n* (%)^c^		
Under 18 years	25 (64.1)	0 (0.0)
18 years or older	14 (35.9)	38 (100.0)
Sex, *n* (%)		
Female	9 (23.1)	33 (86.8)
Male	30 (76.9)	5 (13.2)
Country, *n* (%)		
Germany	1 (2.6)	1 (2.6)
Italy	8 (20.5)	8 (21.1)
Spain	11 (28.2)	10 (26.3)
Turkey	17 (43.6)	17 (44.7)
UK	0 (0.0)	0 (0.0)
US	2 (5.1)	2 (5.3)
Education, *n* (%)		
None	14 (35.9)	1 (2.6)
Primary school	15 (38.5)	2 (5.3)
Secondary school	5 (12.8)	3 (7.9)
High school	2 (5.1)	16 (42.1)
Vocational school	1 (2.6)	6 (15.8)
University education	1 (2.6)	10 (26.3)
Prefer not to say	1 (2.6)	0 (0.0)
Employment status, *n* (%)		
Employed, full time	0 (0.0)	10 (26.3)
Employed, part time	1 (2.6)	16 (42.1)
Self‐employed	0 (0.0)	3 (7.9)
Unemployed	17 (43.6)	3 (7.9)
Student, full time	8 (20.5)	0 (0.0)
Student, part time	1 (2.6)	0 (0.0)
Homemaker	0 (0.0)	6 (15.8)
Unable to work due to GD3	6 (15.4)	0 (0.0)
Other^d^	2 (5.1)	0 (0.0)
Prefer not to say or missing	4 (10.3)	0 (0.0)
Marital status, *n* (%)		
Single	NA	1 (2.6)
Married/partnership	NA	33 (86.8)
Separated/divorced	NA	3 (7.9)
Prefer not to say	NA	1 (2.6)
Relationship to GD3 patient, *n* (%)		
Mother	NA	34 (89.5)
Father	NA	2 (5.3)
Legal guardian	NA	1 (2.6)
Partner	NA	1 (2.6)

*Note:* Marital status of the patients with GD3 was not collected as it was expected most would be children. Relationship to patient with GD3 can only be collected from caregivers.

Abbreviations: GD3, Gaucher disease Type 3; NA, not applicable.

^a^ Data collected through the patient/caregiver survey.

^b^ Informal caregivers are not professional contracted caregivers, for example, family members or friends.

^c^ Only caregivers who were at least 18 years old could participate in the study.

^d^ Free text after selecting “other” indicates that the patients are children.

#### 3.2.2 Other GD3 Related Resource Use

Patient reported resource use associated with GD3 is shown in Table 3. Most patients use health devices or have made adaptations in their homes. The most common were adaptations to the bathroom (*n* = 25, 64.1%), the vehicle (*n* = 21, 53.8%), and bed rails (*n* = 21, 53.8%).

In the past 12 months, patients with GD3 used alternative/complementary therapies to alleviate GD3‐related symptoms; the most frequently reported were consultations with nutritionists (*n* = 25, 64.1%), physiotherapists (*n* = 17, 43.6%), and massage therapists/acupuncturists (*n* = 11, 28.2%).

#### 3.2.3 Caregivers' Characteristics

Caregivers' characteristics collected in the PC survey are reported in Table 4. The PC survey was completed by 38 informal caregivers. Caregivers were predominantly female (*n* = 33, 86.8%) and married or in a partnership (*n* = 33, 86.8%), with most being the patient's mother (*n* = 34, 89.5%). A large majority held at least a high school education (*n* = 32, 84.2%), and their mean (SD) age at the time of the study was 44.16 (9.23) years.

#### 3.2.4 Caregivers' Burden

##### 3.2.4.1 WPAI

Almost three‐quarters of caregivers (73.7%) were employed at the time of completing the survey, and their work was affected by their caregiving, both by missing work (absenteeism; mean [46]: 22.0% [16.1]) and by reduced productivity when working (presenteeism; mean [46]: 63.9% [21.8]). The mean overall work impairment (which combines absenteeism and presenteeism) was 70.3% (SD = 22.0). Across all caregivers (regardless of employment status), the mean activity impairment was 67.6% (SD = 20.6) (see Table 5).

**TABLE 5:** Informal caregivers' burden.

Informal caregivers' WPAI questionnaire	
Currently employed, *n* (%)	
Yes	28 (73.7)
No	10 (26.3)
WPAI scores: Caregiver currently employed (*n* = 28); mean (SD)	
Absenteeism^a^	22.0 (16.1)
Presenteeism^b^	63.9 (21.8)
Overall work impairment^c^	70.3 (22.0)
WPAI scores: All informal caregivers (*n* = 38); mean (SD)	
Activity impairment^d^	67.6 (20.6)
Ever stopped working due to GD3 caregiving duties, *n* (%)	
Yes	16 (42.1)
Hours of informal care per week^e^; mean (SD)	
Hours of informal care per week	63.29 (40.15)
CarerQoL‐7D; mean (SD)	
Index (UK utility values)^f^	67.04 (15.78)
VAS [CarerQoL‐VAS]^g^	5.34 (1.86)
EQ‐5D‐5L; mean (SD)	
Index (English value set)^h^	0.85 (0.10)
VAS [EQ‐5D‐5L‐VAS]^i^	77.29 (15.05)

Abbreviations: CarerQoL‐7D, CareQoL seven‐dimension; EQ‐5D‐5L, five‐level EuroQol five dimension; GD3, Gaucher disease Type 3; SD, standard deviation; VAS, visual analog scale; WPAI, Work Productivity Activity Impairment.

^a^ Indicates proportion of working hours missed due to GD3 caregiving. Range: 0 (no working hours missed) to 100 (all working hours missed).

^b^ Range: 0 (no effect on work productivity) to 100 (no productivity at work).

^c^ Combines absenteeism and presenteeism into a single score.

^d^ Range 0 (no problems) to 100 (completely prevented doing daily activities).

^e^ Note: Contracted care is only reported for three patients, and the hours per week range between 8 and 25.

^f^ Score ranges from 0 (worst imaginable caregiving situation) to 100 (best imaginable caregiving situation).

^g^ How happy do you feel at the moment (0 = Completely unhappy; 10 = Completely happy).

^h^ Score ranges from 0 (health state equivalent to death) to 1 (perfect health).

^i^ How good or bad is your health today (0 = the worst health you can imagine; 100 = the best health you can imagine).

##### 3.2.4.2 Stopping Work due to Caregiving Responsibilities

While the majority of caregivers were employed at the time of the survey, 42.1% (*n* = 16/38) reported having stopped working at some point as a result of their GD3 caregiving duties (see Table 5).

##### 3.2.4.3 Hours of Informal Care

On average, caregivers provided 63.29 h (SD = 40.15) of informal care per week (Table 5), exceeding a standard full‐time workweek, with some caregivers providing care 24/7.

##### 3.2.4.4 CarerQoL‐7D

Results from the CarerQoL‐7D suggest that caregivers experienced a notable burden related to their caregiving responsibilities (Table 5), with a mean (SD) index of 67.04 (15.78) and VAS of 5.34 (1.86). As shown in Figure 2, while most caregivers reported experiencing fulfillment in their caregiving role, over 80% also indicated some or significant challenges related to mental health, balancing caregiving with daily activities, and financial strain. Additionally, nearly 70% reported experiencing physical health problems associated with their caregiving responsibilities.

**FIGURE 2:**
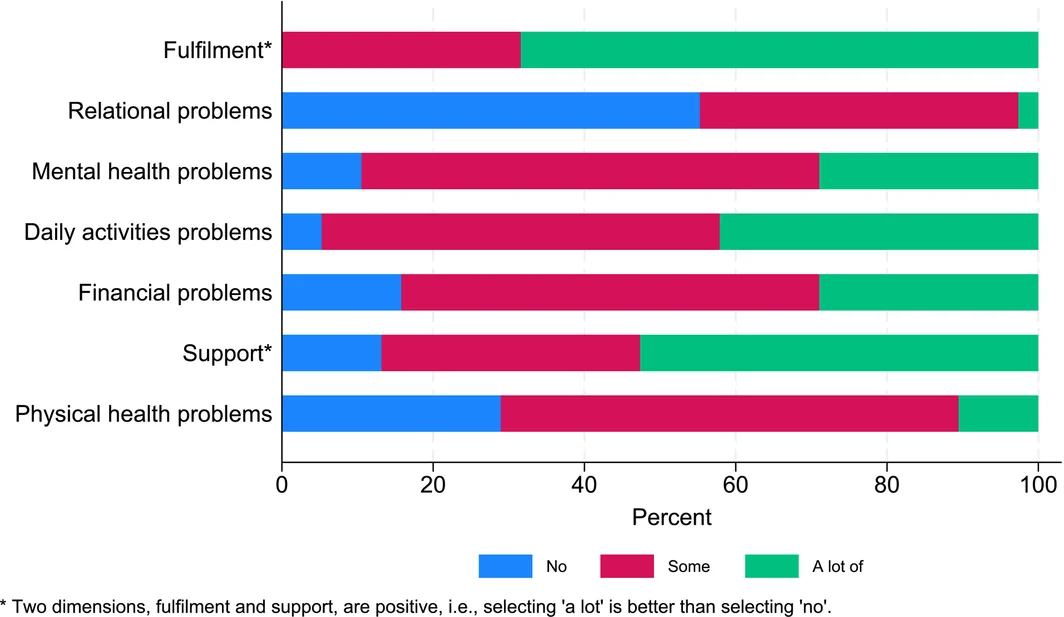
Informal caregivers' CarerQoL‐7D responses for each dimension.

##### 3.2.4.5 EQ‐5D‐5L

Results by EQ‐5D‐5L (Figure 3) show that caregivers reported good mobility and selfcare (over 90% reported no problems for either). However, they reported a moderate to high burden in the pain domain (almost 60% reported slight or moderate problems). The highest burden was reported in the anxiety/depression domain: over 80% of caregivers reported problems, nearly 40% reported moderate problems, and around 5% reported severe or extreme problems. The combination of all these domains resulted in an index (Table 5) averaging 0.85 (SD = 0.10) and the VAS averaging 77.29 (SD = 15.05).

**FIGURE 3:**
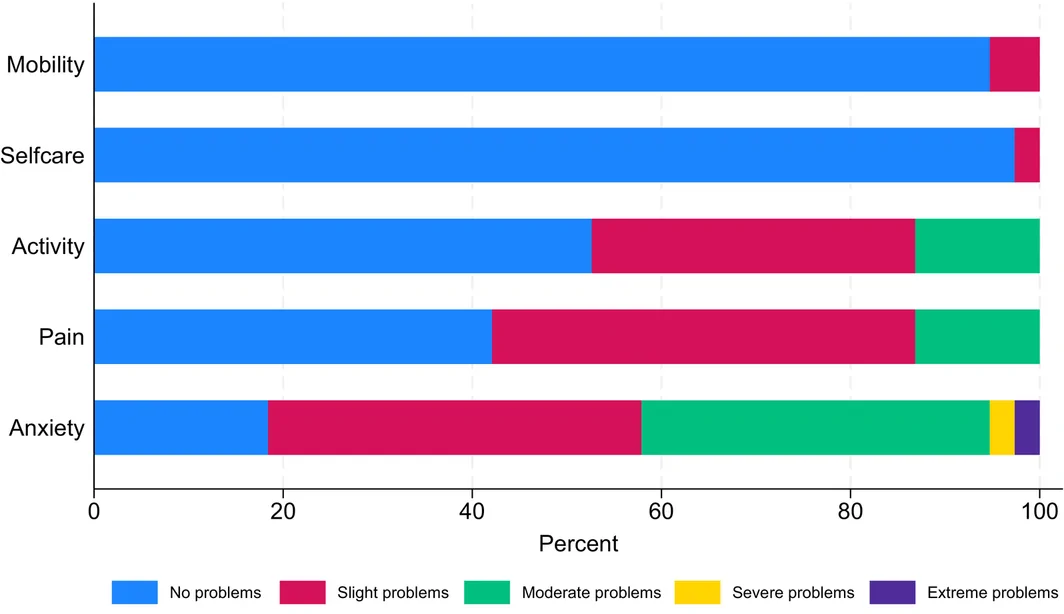
Informal caregivers' EQ‐5D‐5L responses (levels) for each dimension.

## 4 Discussion

The study highlights the burden of GD3 from multiple perspectives, including effects on patients (including clinical burden), caregivers, healthcare systems, and broader societal impacts using real‐world data collected from six countries.

In this study, the mean age of patients at the time of data collection was 17.01 years (SD = 9.03), which aligns with previously published data, where average ages of GD3 cohorts ranged from 12.4 [47] to 26 years [12, 13, 15]. A recent analysis of the French Gaucher registry reported a median age of 16 years for patients with GD3 (*Q*1, *Q*3:10–31) [14], closely matching our findings (median: 16 years; *Q*1, *Q*3: 10–23).

The mean age at diagnosis reported by clinicians in this study was 8.16 years (SD = 6.36), with a median of 6 years (*Q*1, *Q*3: 3–13). This is higher than the average age reported in other studies [1, 12, 13, 14, 15, 16, 17, 18, 31, 47, 48]. Several factors may account for this difference. First, age at diagnosis may be influenced by the severity of the clinical phenotype. Almost half of the patients with available genotype data in this study have the genotype p.L483P/p.L483P, consistent with a previous GD3 study reporting a high prevalence of this variant (67%) [13], which is associated with variable manifestation [5, 12, 49], and almost one‐fifth carried a genotype (p.L483P/p.R502C) which shows an attenuated phenotype [12]. In addition, one‐fifth of the patients were from Turkey, where a milder phenotype is common, typically presenting between ages 5 and 7 years, often resulting in diagnostic delays of up to 2 years [50, 51]. Early differentiation between GD1 and GD3 is challenging due to phenotypic variability and the delayed onset of CNS symptoms, which may lead to misclassification or delayed recognition [13]. Other potential contributors to delayed diagnosis include limited disease awareness and differences in diagnostic infrastructure across countries and over time [52]. Earlier diagnosis has become more likely in recent years due to improved awareness and testing availability, whereas older patients may have experienced longer diagnostic delays [14, 53, 54].

Regarding the clinical burden, patients with GD3 experienced a range of neurosystemic manifestations, both at the age they first presented with symptoms and at the time of data collection.

Many of the manifestations first reported remained present at the index date, indicating an ongoing clinical burden and highlighting the chronic, multisystem nature of GD3 [48].

The time from initial clinical manifestations to GD3 diagnosis—approximately 2 years for somatic symptoms and 1 year for CNS symptoms—was consistent with findings from previous studies [15, 47].

Regarding health resource use, in the 2 years prior to the index date, patients with GD3 underwent multiple tests (e.g., blood test, neurological examination), and almost half of them had inpatient hospital admissions; most of the admissions were due to CNS and bone/joint complications. These frequent healthcare interactions highlight the significant burden GD3 places on healthcare systems in terms of resource utilization and associated costs.

The study results highlight the considerable impact of GD3 on patients' daily activities. Clinician‐reported data indicated that over half of the patients experienced problems with daily activities, such as falls or problems with personal care, likely related to the use of health devices or making home alterations, as reported by most patients. Patients with GD3 also reported using alternative and complementary therapies to manage their condition; however, these treatments can impose a significant financial burden, particularly given the variability in insurance coverage across countries. Given the limited GD3‐specific evidence currently available, further research is needed to deepen our understanding of how GD3 affects the daily lives and well‐being of patients.

Our results also show that caregivers of patients with GD3 face a substantial burden that impacts multiple aspects of their lives, which is consistent with findings from a Japanese study reporting that caregivers of individuals with GD3 experience greater burden than those caring for patients with GD1 [55]. Using the WPAI tool, our findings show the substantial impact of GD3 on both work productivity and daily activities of caregivers. While most caregivers are employed at the time of the survey, caregiving responsibilities interfere with their ability to work, with a mean overall work impairment of 70%, and around two in five (42.1%) had to stop working because of caregiving at some point in the past. For comparison, overall work impairment among caregivers of individuals with Duchenne muscular dystrophy (DMD)—a rare genetic disorder affecting children and requiring long‐term care—has been estimated to range between 20% and 29% [56], considerably lower than observed in caregivers of patients with GD3. Additionally, caregivers of patients with GD3 also report dedicating large amounts of hours to caregiving, on average, more than the equivalent of a full‐time working week. Some even describe caregiving as a 24/7 responsibility. These results highlight the considerable impact of GD3 on caregiver productivity and daily life, providing a quantifiable measure of its societal burden.

In terms of HRQoL, caregivers of patients with GD3 reported average EQ‐5D‐5L index scores comparable with those observed in the general population. The average score was 0.85, which falls within the range reported for women aged 30–64—the demographic that makes up the majority of caregivers—in the 2017 and 2018 Health Survey for England (0.776–0.869) [57]. However, a closer examination of the EQ‐5D dimensions reveals that nearly 40% of caregivers reported moderate problems with anxiety and depression, and approximately 5% reported severe or extreme problems. It is important to note that the EQ‐5D may not fully capture the caregiving burden, as it primarily focuses on physical health [58] and relies on a standard definition of “usual activities,” which may not reflect the adapted routines of caregivers [59].

To address this gap, the caregiver‐specific CarerQoL‐7D instrument was used and indicated a notable burden, with an average index score of 67 and a VAS score of 5.34. This VAS is notably lower than that for caregivers of patients with Pompe disease—a rare genetic disorder affecting children and requiring long‐term caregiving—in the Netherlands, whose average was 7.2 [60]. Dimension‐level results show that while many caregivers reported feelings of fulfillment and support, most also reported challenges related to mental health, daily functioning, and financial strain.

Overall, the results of this study indicate that the burden of GD3 experienced by both patients and caregivers has different forms, affecting different aspects of their lives. Patients experience clinical burden (e.g., multiple neurosystemic manifestations), resource burden (e.g., alternative therapies that might not be covered by insurance), and the impact of GD3 on daily activities. Patients with GD3 also have high use of healthcare resources, including frequent hospitalizations, which contribute to a considerable burden on healthcare systems. Caregivers devote a large number of hours to caregiving, which affects their work productivity and their HRQoL, particularly their mental health. This burden is expected to increase over time, as a Japanese study identified a positive association between caregiver burden scores and the duration of caregiving among caregivers of patients with GD3 [55].

Our findings have important clinical and policy implications. The considerable and multidimensional burden experienced by caregivers highlights the need for early recognition and systematic assessment of caregiver burden to facilitate timely and effective support interventions [55]. Coordinated multidisciplinary care, including structured care pathways and integrated psychosocial support for patients and their families, may help alleviate burden and improve caregiver quality of life [61].

### 4.1 Limitations

Study findings should be interpreted in light of some limitations. Although clinicians participating in this study voluntarily joined a research panel, they might not be representative of all clinicians practicing in each country, and therefore, their patients might not be representative of GD3 in each country. Similarly, not all patients/caregivers decided to complete the PC survey. Therefore, those completing it might not be representative of the patients/caregivers in each country. The surveys relied on participants' (clinicians, patients, and caregivers) ability to accurately recall past events, circumstances, and experiences. As with any self‐reported data, imperfect recall may have affected some responses.

Health resource use associated with GD3 was collected for the 24‐month period prior to index. This period helps to capture some of the year‐to‐year variability in patients' resource use but comes at a risk of respondent fatigue and imperfect recall. Patients might be receiving Enzyme Replacement Therapy (ERT) and/or Substrate Replacement Therapy (SRT), which could influence their outcomes—such as clinical manifestations—compared with those not receiving treatment.

The study includes data from six countries; there might be differences in the data available due to local practices and in the definitions of some of the variables captured in the surveys.

## 5 Conclusions

This study is among the first to document the real‐world burden of GD3. It contributes to an underserved research area by providing an up‐to‐date, multinational perspective on the clinical history and burden of GD3 for patients, caregivers, families, healthcare systems, and society.

Patients bear a significant clinical and functional burden, characterized by a wide spectrum of neurosystemic manifestations, with most diagnosed in early childhood. They also incur high healthcare resource use, including frequent hospitalization, reliance on health devices, and the need for home modifications.

Caregivers experience high levels of work impairment, emotional strain, and reduced quality of life, particularly in mental health. These findings underscore the need for timely diagnosis, coordination of multidisciplinary and holistic care approaches, and greater psychosocial support for caregivers. Further research, including PROs, is essential to fully understand the impact of GD3.

## Author Contributions

All authors contributed to the study conception, data interpretation, and manuscript preparation. I.R.S. carried out statistical analyses. I.R.S., L.D., and J.M.P. drafted the first version of the manuscript. All authors read and approved the final manuscript. E.M. and F.S.E. had oversight of the study and provided critical review of the manuscript. L.D. serves as the guarantor for this article, taking full responsibility for the work and/or the conduct of the study, had full access to the data, and controlled the decision to submit the manuscript for publication.

## Funding

This study was funded by Sanofi. The authors confirm independence from the sponsor; the content of the article has not been influenced by the sponsor.

## Ethics Statement

This study was reviewed by the WCG Institutional Review Board and received an exemption determination.

## Consent

Electronic informed consent was obtained from all study participants or, where applicable, their legal representatives.

## Conflicts of Interest

**E.M**. received research grants, speakers' fees, and consulting honoraria from Sanofi, Orphazyme, Takeda, Cyclo Therapeutics, Idorsia, JCR, Denali, Amicus, Spurs Therapeutics, Prevail, and Avrobio. **S.S**. received research grants, speakers' fees, and consulting honoraria from Sanofi. **A.D.F**. declares no conflicts of interest. **A.D**. has received consulting and speakers' fees from Sanofi. **L.D**., **M.G.P**., and **J.P**. are employees of Sanofi and may hold stocks and/or stock options in the company. **R.P.‐J**. was an employee of Sanofi at the time the research was conducted. **I.R.S**. is an employee of Prime HCD; Sanofi hired Prime HCD to conduct this study. **F.S.E**. received research grants, speakers' fees, and consulting honoraria from Sanofi. **T.C.‐H**. IGA received an annual grant from Sanofi to support the IGA's Global Work Program in 2024 and 2025. **T.C.‐H**. received a consulting honorarium from Sanofi.

## Supporting information


**Table S1:** Study participation.
**Table S2:** Patient's age when clinical manifestation first appeared.

## Data Availability

Qualified researchers may request access to patient‐level data and related study documents, including the study protocol, statistical analysis plan, and dataset specifications. Patient‐level data will be anonymized, and study documents will be redacted to protect the privacy of study participants. Further details on Sanofi's data sharing criteria, eligible studies, and process for requesting access can be found at: https://www.vivli.org/.
