# Supplementary material for: Clinical History and Burden of Gaucher Disease Type 3: A Cross‐Sectional Multinational Study
Source: JIMD Rep. 2026 Oct 30;67(6):e70127. doi: 10.1002/jmd2.70127 (PMC13722778; doi:10.1002/jmd2.70127)
Supplement: Supplementary file 1 [file JMD2-67-e70127-s001.docx]

**Table S1. Study participation**

| **Total number of clinicians** | 34 |
| --- | --- |
| **Clinicians, by country; n (%)** |  |
| Germany | 3 (8.8) |
| Italy | 5 (14.7) |
| Spain | 7 (20.6) |
| Turkey | 9 (26.5) |
| UK | 3 (8.8) |
| USA | 7 (20.6) |
| **Clinicians, by specialty; n (%)** |  |
| Cardiology | 1 (2.9) |
| Genetics | 5 (14.7) |
| Hemato-oncology | 4 (11.8) |
| Hematology | 6 (17.7) |
| Hepatology | 1 (2.9) |
| Inherited Metabolic Disorders | 2 (5.9) |
| Internal medicine | 4 (11.8) |
| Neurology | 5 (14.7) |
| Orthopedic Medicine | 1 (2.9) |
| Pediatrics | 4 (11.8) |
| Other (Nephrology) | 1 (2.9) |

**Table S2. Patient’s age when clinical manifestation first appeared**

| **Clinical manifestation** | **mean (SD)/median (25th, 75th percentile)** |
| --- | --- |
| 1. Cognitive impairment (n=26) | 4.46 (2.92) / 4 (3; 5) |
| 2. Developmental delay (n=35) | 4.69 (3.22) / 4 (3; 5) |
| 3. Developmental regression (n=5) | 5.00 (5.05) / 2 (2; 7) |
| 4. Ataxia (n=22) | 7.86 (5.04) / 6.5 (4; 10) |
| 4.1 Gait/walking (n=11) | 6.91 (4.16) / 7 (4; 8) |
| 4.1.1 Abnormal no support (n=6) | 6.83 (3.54) / 6 (4; 10) |
| 4.1.2 Abnormal with support (n=7) | 7.86 (5.58) / 5 (5; 8) |
| 4.1.3 Unable to walk (wheelchair dependent) (n=3) | 10.67 (2.08) / 10 (9; 13) |
| 4.2 Balance (n=15) | 7.33 (4.88) / 6 (4; 8) |
| 4.3 Speech (n=13) | 6.69 (3.71) / 6 (5; 7) |
| 4.3.1 Sometimes difficult to understand (n=8) | 6.63 (4.84) / 5 (4; 7) |
| 4.3.2 Most of the time unintelligible (n=7) | 6.71 (1.11) / 7 (6; 8) |
| 4.3.3 Not able to communicate (n=0) | No observations |
| 5. Swallowing difficulties (n=17) | 7.06 (4.58) / 6 (5; 7) |
| 5.1 Choking (n=9) | 6.44 (4.45) / 5 (4; 6) |
| 5.2 Need for a special diet/food texture (n=13) | 5.15 (1.41) / 5 (4; 6) |
| 6. Tremor(n=5) | 11.40 (6.62) / 8 (6; 17) |
| 7. Myoclonus (n=2) | 9.50 (6.36) / 9.5 (5; 14) |
| 8. Seizures/epilepsy (n=29) | 8.72 (5.92) / 7 (5; 12) |
| 9. Hearing loss (n=4) | 6.00 (3.37) / 6 (3.5; 8.5) |
| 10. Weakness, muscle hypotonia (n=24) | 5.21 (4.23) / 4 (3; 6) |
| SD, standard deviation. | |
